# Decoding the Plasticity of Cancer‐Associated Fibroblasts: Mechanistic Insights and Precision Targeting Strategies in Gastric Cancer Progression and Therapeutic Resistance

**DOI:** 10.1111/cpr.70094

**Published:** 2025-07-23

**Authors:** Shiyang Deng, Yong zhen Chen, Jiang Du

**Affiliations:** ^1^ Department of Pathology The First Affiliated Hospital of China Medical University Shenyang China; ^2^ Department of Pathology, College of Basic Medical Science China Medical University China

To the Editor,

Gastric cancer (GC), the fourth leading cause of global cancer mortality, remains clinically problematic despite advances in multimodal treatments including surgery, chemotherapy, and immunotherapy. The consistently poor outcome in advanced stages needs new treatment approaches. Cancer‐associated fibroblasts (CAFs) have emerged as important regulators of gastric tumour growth by dynamic interactions inside the tumour microenvironment (TME). These heterogeneous stromal elements, which are produced from several precursors, show continuous phenotypic evolution during carcinogenesis. CAFs mediate tumour‐stroma crosstalk by extracellular matrix remodelling, paracrine signalling via growth factors/cytokines, and extracellular vesicle communication. Notably, CAFs and malignant cells show therapeutic pressure‐driven co‐evolution, and CAF‐derived IL‐6/IL‐8 activate STAT3/NF‐κB pathways to promote metabolic reprogramming while also creating drug‐resistant ECM barriers. We aim to provide a transformative framework for understanding CAFs biology in gastric oncology, analysing molecular mechanisms of invasion, explaining tumour‐CAF co‐evolution dynamics, and mapping resistance‐related regulatory networks.

## 1 Heterogeneous Origin and Functional Diversity of CAFs

Cancer‐associated fibroblasts (CAFs) originate from diverse cellular precursors within the tumour microenvironment, including activated fibroblasts, endothelial cells, epithelial cells undergoing epithelial‐mesenchymal transition (EMT), mesenchymal/haematopoietic stem cells [1], adipocytes, pericytes, and stellate cells. Their transformation is governed by growth factors (e.g., TGF‐β, PDGF, FGF, HGF), inflammatory cytokines (e.g., TNF, IL‐6), and genetic alterations such as RAS/Myc oncogenic mutations or p53/PTEN tumour suppressor inactivation. CAFs heterogeneity stems from their multipotent origins, reflected in divergent surface markers and functional profiles. Single‐cell analyses have resolved six functionally distinct CAF subclasses: three dominant subtypes—myofibroblastic (CAF‐myo), inflammatory (CAF‐infla), and adipose‐derived (CAF‐adi)—and three minor subsets including endothelial‐mesenchymal transition (CAF‐EndMT), peripheral nerve‐like (CAF‐PN), and antigen‐presenting (CAF‐ap) variants. Functionally, CAF subpopulations differentially regulate tumour progression through cancer cell proliferation, angiogenesis, ECM remodelling, and immunosuppression [2] (Table 1).

**TABLE 1:** Classification, function, marker and clinical application of tumour‐related fibroblasts (CAFs) subgroups in Gastric Cancer.

Origin/subset type	Functional characteristics	Specific markers	Clinical significance in gastric cancer	Personalised therapeutic strategies
Mesenchymal stem cell‐derived CAFs	Promotes GC invasion/metastasis, immunosuppression (secretes TGF‐β, IL‐10), induces chemotherapy resistance	FAP, α ‐SMA, PDGFRβ	FAP overexpression correlates with reduced overall survival; FAP‐targeted PET imaging for GC staging	FAP‐directed CAR‐T cell therapy (clinical trials), FAP inhibitors (Talabostat) combined with chemotherapy
Normal fibroblast‐Transformed CAFs	Stroma remodelling (secretes MMP2/9, Collagen XI), pro‐angiogenic (VEGF‐A)	S100A4 (FSP1), POSTN (Periostin)	S100A4^+^ CAFs linked to peritoneal metastasis; POSTN as a biomarker for poor prognosis	Anti‐POSTN mAb (DS‐6016a) or MMP inhibitors (Andecaliximab) combined with anti‐ angiogenic agents
Adipocyte‐derived C AFs	Lipid metabolic reprogramming (secretes FABP4), pro‐inflammatory microenvironment (IL‐6/IL‐8)	FABP4, ADIPOQ, Leptin receptor	FABP4 + CAFs associated with cisplatin resistance in obese GC patients	Targeting fatty acid oxidation (Etomoxir) or leptin receptor inhibitors (Pegteptin)
Endothelial‐mesenchymal transition CAFs	Facilitates vasculogenic mimicry, immune evasion (upregulates PD‐L1/PD‐L2)	Endosialin (CD248), Vimentin, CD31—	Endosialin + CAFs correlate with anti‐PD‐1 therapy resistance	Anti‐Endosialin antibody (Ontuxizumab) combined with immune checkpoint inhibitors
Pericyte‐derived CAFs	Enhances tumour mechanical barrier (ECM stiffness), confers radiotherapy resistance	NG2, PDGFR β, α‐SMA (weak expression)	NG2+ CAFs linked to post‐radiotherapy local recurrence	Small‐molecule PDGFRβ inhibitors (Imatinib) combined with radiotherapy

## 2 Mechanism of Dynamic Remodelling of ECM by CAFs

The extracellular matrix (ECM) is a complex network structure maintained in a dynamic equilibrium, primarily composed of proteins, polysaccharides, and hydrated gels. CAFs contribute to the remodelling of the tumour microenvironment at multiple levels, including the regulation of ECM stiffness, synthesis of matrix proteins, and degradation of ECM components, through the secretion of various biologically active molecules, thereby promoting the invasive process of tumour cells. Research has shown that CAFs upregulate the expression of HAPLN1 through the TGF‐β1/Smad2/3 signalling pathway to regulate the stiffness level of ECM, change the biomechanical properties of ECM [3], create an invasive pathway for gastric cancer cells, and reduce the physical barrier, thus significantly enhancing its invasive potential. Emerging evidence confirms that cancer‐associated fibroblasts (CAFs) facilitate collective tumour cell invasion through extracellular matrix (ECM) reconfiguration, creating distinct migratory trajectories. CAF‐derived cytokines and matrix remodelling enzymes regulate the physical and chemical properties of the tumour microenvironment, particularly by increasing ECM stiffness to increase the invasiveness of cancer cells [4].

## 3 Bidirectional Signalling Regulation Between CAFs and Gastric Cancer Cells

CAFs secrete TGF‐β1, IL‐1β, and CXCL12 to drive gastric cancer progression via SMAD/NF‐κB signalling. TGF‐β1 activates SMAD‐dependent EMT, enhancing tumour cell migration/invasion, while IL‐1β promotes proliferation through NF‐κB activation. CXCL12‐CXCR4 interactions inhibit apoptosis and stimulate growth. Reciprocally, gastric cancer cells reprogram CAFs: TGF‐β1 induces Smad2‐mediated CAFs differentiation from BMSCs, while NF‐κB‐driven INHBB upregulation converts normal fibroblasts to CAFs. This bidirectional cytokine network perpetuates tumour‐stroma crosstalk, synergistically enhancing both cancer cell aggressiveness and CAFs activation. CAFs further strengthen their interactions with gastric cancer cells by precisely altering the mechanical properties of the gastric cancer microenvironment. Specific types of collagen secreted by CAFs activate mechanoreceptors in gastric cancer cells by increasing the stiffness of the microenvironment and upregulate the expression of CTGF, which not only enhances the infiltration capacity of CAFs but also promotes the continuous secretion of collagen to form a NF‐κB‐PIEZO1‐YAP1‐CTGF positive‐feedback loop. This mechanism reveals the decisive role of mechanosensitive signalling in the gastric cancer microenvironment in tumour progression. Additionally, *H. pylori* ‐induced inflammatory responses further enhance the synergistic interaction between gastric cancer cells and CAFs through the PIEZO1 and NF‐κB signalling pathways [5]. The interactions between CAFs and gastric cancer cells also exhibit highly specific regulatory features. Studies have shown that interleukins (IL‐6, IL‐8, IL‐11, etc.) derived from CAFs play a central role in gastric cancer progression, and more critically, they create a microenvironment precisely mediated by protein modifications that continuously stimulate the production of IL‐6 and IL‐8 by stromal cells. PKM2, which is highly expressed in the microenvironment, provides a specific positive‐feedback regulatory mechanism. By mediating the acetylation modification of the P65 protein within CAFs, it maintains the continuous activation of the NF‐κB signalling pathway [6], significantly enhancing the ability of CAFs to secrete inflammatory factors such as IL‐6 and IL‐8 and further promoting the proliferation and invasion of gastric cancer cells [7].

## 4 Interactions Between CAFs and Immune Cells

CAFs originate from various cell types within the tumour microenvironment (TME) and form an intricate network of interactions with multiple biological components of this environment [8]. Through the secretion of cytokines, chemokines, and metabolites, CAFs finely regulate the tumour immune microenvironment (TIME), which subsequently influences tumour progression. The advent of spatially resolved transcriptomics (SRT) has revolutionised our understanding of the interactions between different cells within the tumour microenvironment (TME), featuring the analysis of genome‐wide gene expression while preserving spatial structure. This technology allows precise mapping of the molecular crosstalk mechanisms between CAFs and immune cells, revealing the contextually relevant signalling networks that drive tumour progression and immune evasion. Recent studies using the SRT platform have identified spatially restricted CAFs subpopulations with distinct immunomodulatory functions. For example, inflammatory CAFs (iCAFs) localised in the vicinity of CD8^+^ T‐cell‐enriched regions of gastric cancer secrete CXCL12 and TGF‐β, which recruit regulatory T cells (Tregs) and polarise macrophages to an immunosuppressive M2 phenotype, and ligand‐receptor pairs (e.g., CXCL12‐CXCR4, TGFB1‐ TGFBR2) as evidenced by co‐localisation analysis. In contrast, myofibroblast CAFs (myCAFs) [9], which form the stromal barrier surrounding the tumour, up‐regulate extracellular matrix (ECM) genes (e.g., COL1A1, FN1) and physically repel cytotoxic T lymphocytes (CTLs) [10].

## 5 Phenotypic Plasticity and Molecular Interactions of CAFs in Chemotherapy Resistance

Chemotherapy‐induced stress promotes phenotypic plasticity and metabolic reprogramming in CAFs, characterised by altered expression of enzymes like HK2 and LDHA. This remodelling fuels tumour growth via lactate secretion and activates downstream signalling through metabolic‐epigenetic pathways. Metabolomic studies reveal that CAF‐derived lactate upregulates P‐glycoprotein through HIF‐1α activation, establishing drug‐resistant phenotypes [11]. The metabolic‐epigenetic axis facilitates energy transfer to malignant cells while stabilising chemoresistance mechanisms, creating a microenvironment conducive to treatment evasionAt the level of metabolic reprogramming, a highly complex metabolic interaction network is constructed between CAFs and gastric cancer cells. This metabolic network transcends a simple pattern of single metabolite exchange and forms a systematic metabolic‐signal transduction‐epigenetic regulation axis. Specifically, highly activated CAFs generate large amounts of lactate through the glycolytic pathway, which not only serves as a preferred energetic substrate for drug‐resistant gastric cancer cells to meet their ATP demand but also promotes the expression of PD‐L1 through a dual mechanism: lactate directly up‐regulates PD‐L1 transcription by activating the NF‐κB pathway and induces modification of the PD‐L1 promoter through histone lactate modification [12], remodelling the chromatin structure to form an epigenetically activated state. Further analysis confirmed that this metabolic‐epigenetic regulatory axis enhances the level of H3K27ac modification in the P‐glycoprotein promoter region by recruiting the CBP/p300 acetyltransferase complex in a HIF‐1α‐dependent manner, thereby promoting MDR1 gene expression and maintaining a stable drug‐resistant phenotype [13].

## 6 CAFs‐Mediated Microenvironmental Heterogeneity and Tumour Stem Cell Maintenance

Recent studies have systematically revealed that resistant clones of chemotherapy residues are preferentially localised in regions with high stromal stiffness (> 15 kPa). In these regions, Collagen I, upregulated by ECM remodelling, specifically binds to the DDR2 receptor, activating the NF‐κB signalling pathway and inducing upregulation of PD‐L1 expression. CAFs in this region show high expression of TGF‐β and VEGF‐A, thereby constructing a unique local immunosuppressive microenvironment [14]. This microenvironment heterogeneity exhibits more pronounced features in residual tumours after chemotherapy, suggesting that it may play a key role in the selective amplification of drug‐resistant clones. Tumour stem cells (CSCs), central drivers of chemoresistance and relapse in gastric cancer, are finely regulated by CAFs, which maintain the CSC population and promote the transformation of non‐stem cells into CSCs through the construction of a multilayered molecular network, a process closely linked to the formation of chemoresistance. CAFs regulate the CSC population through multiple mechanisms [1], including ligand‐receptor interactions, signalling cascades, and epigenetic modifications. At the level of the signalling network, neuromodulatory protein 1 (NRG1) secreted by CAFs plays a key role in maintaining the properties of CSCs. In‐depth studies have shown that NRG1 activates the downstream PI3K/AKT and IKK/NF‐κB signalling pathways by binding to ErbB3/ErbB4 heterodimeric receptors on the surface of CSCs. Activated NF‐κB not only directly binds to the promoter and enhancer regions of core stemness genes such as Oct4, Sox2, and Nanog, but also alters the chromatin accessibility of these regions by recruiting the SWI/SNF chromatin remodelling complex. Remarkably, under long‐term chemotherapeutic pressure [7], this epigenetic reprogramming exhibits significant temporal specificity, undergoing a dynamic process from transient activation to stable expression, ultimately resulting in a stable mechanism for maintaining the stemness phenotype [15].

## 7 Multilevel Regulatory Mechanisms of CAFs Mediating Immunotherapy Resistance in Gastric Cancer

**FIGURE 1:**
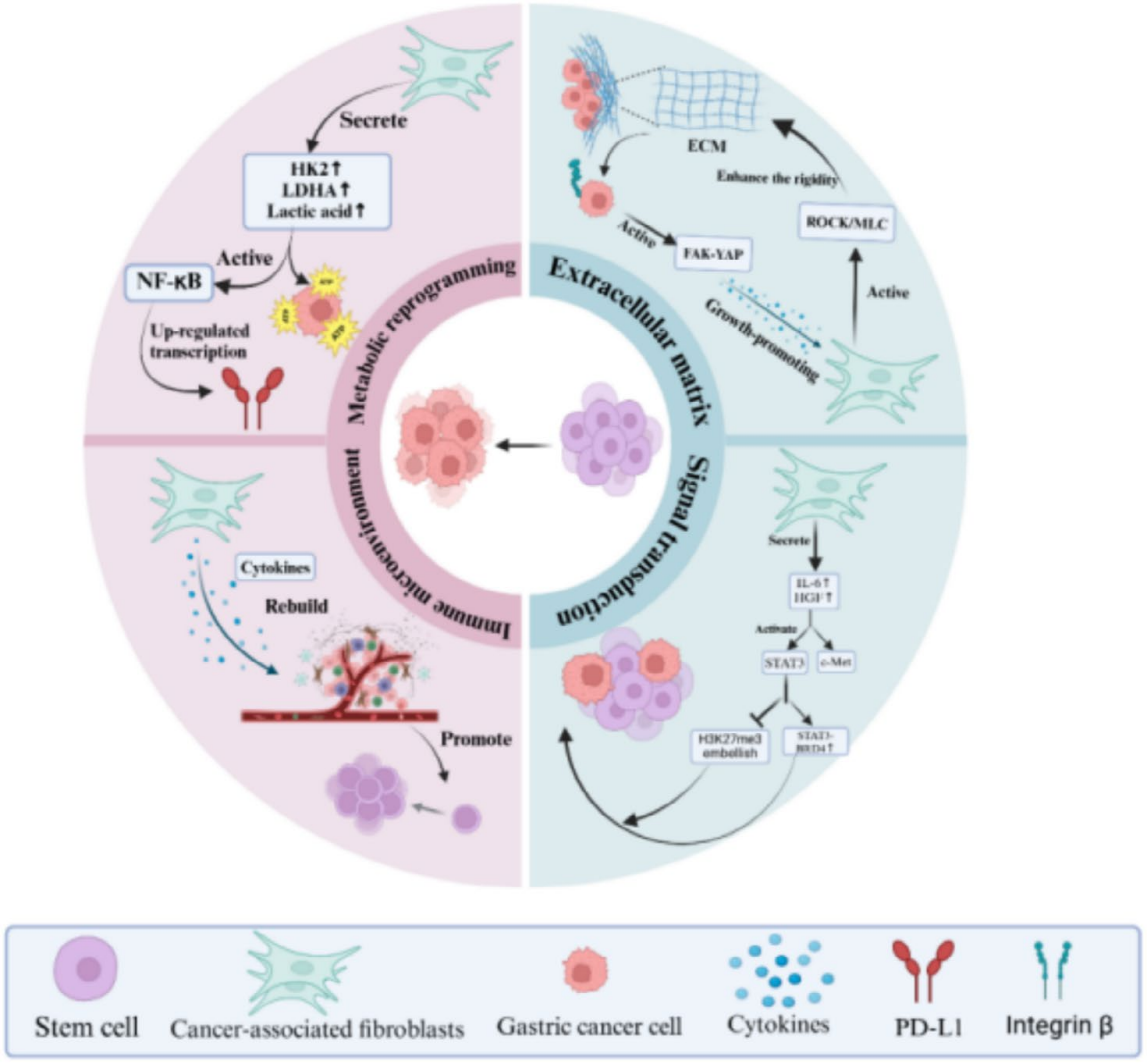
Molecular mechanisms of tumour microenvironment cross‐talk and metabolic reprogramming in gastric cancer.

Research has revealed that CAFs mediate immunotherapy resistance through multiple mechanisms, including ECM remodelling, metabolic network regulation, and immunosuppressive microenvironment formation. Regarding ECM remodelling, CAFs positioned at the tumour‐infiltrating front exhibit high CXCL12, TGF‐β, and CCL2 expression, forming an “immune‐repellent” microenvironment that blocks effector T‐cell infiltration into the tumour core. Metabolically, CAFs enhance glycolysis in tumour cells via LOX and the TGFβ/IGF1 pathway, and lactate accumulation upregulates PD‐L1 and enhances STAT3 activity [16], promoting immunosuppression. CAFs also induce arginine depletion, inhibiting T cells and promoting Treg differentiation. CAF‐derived exosomes enriched in KDM5B reduce tumour cell immunogenicity through epigenetic modifications. Inhibiting ESCRT function reduces KDM5B secretion, enhancing immunotherapy efficacy. Additionally, TGF‐β signalling in CAFs modulates the expression of immunosuppressive genes, maintaining the immunosuppressive microenvironment. These findings highlight the multifaceted role of CAFs in immunotherapy resistance [17] (Figure 1).

## 8 Therapeutic Strategies for Targeting CAFs

CAFs are a crucial component of the tumour microenvironment in GC, influencing tumour invasiveness and drug resistance through secreted factors and signalling pathways. Specifically, They secrete cytokines like IL‐6, IL‐8, TGF‐β1, and CXCL12, driving GC cell invasion and metastasis. Therefore, targeting these pathways to reconfigure the tumour microenvironment and boost chemosensitivity offers promising therapeutic approaches [18]. For instance, IL‐6 activates the JAK1‐STAT3 pathway in GC cells, contributing to chemoresistance. In this regard, tocilizumab, an IL‐6R monoclonal antibody, effectively blocks IL‐6 signalling, promoting cancer cell apoptosis. Similarly, vitamin D reverses IL‐8‐mediated oxaliplatin resistance by inhibiting the PI3K/Akt pathway and is significantly linked to lower cancer risk and improved prognosis. Moreover, TGF‐β1 induces CAFs to secrete IGFBP7, promoting tumour‐associated macrophage polarisation and GC metastasis. In response, trinilast, an anti‐fibrotic agent, suppresses fibroblast proliferation by inhibiting TGF‐β1 signalling, reducing GC cell invasiveness. Additionally, CXCR4 antagonists like AMD3100 and motixafortide (CL‐8040) inhibit the CXCL12‐CXCR4 axis, suppressing GC cell invasion and metastasis. Furthermore, microRNAs (miRNAs) also play a pivotal role in GC progression. Targeting specific miRNAs, such as miR‐149, can inhibit GC cell invasion and metastasis. Lastly, tumour vascularization is a critical process that facilitates the re‐establishment of nutrient supply, thereby supporting tumour progression. Consequently, anti‐angiogenic therapies targeting VEGF, such as ramucirumab, have shown significant efficacy in clinical trials. Cancer immunotherapy targeting CAFs markers like FAP, α‐smooth muscle actin (α‐SMA), and platelet‐derived growth factor receptor (PDGFR) can deplete CAFs and enhance anti‐tumour immunity. FAP‐directed therapies enhance anti‐cancer immune responses. Low‐immunogenic FAP‐CAR T cells (UCAR‐T cells) and vaccines like SynCon FAP has effectively depleted CAFs, mitigated tumour infiltration, and enhanced T cell immunity. Gene editing technology offers a solution to effectively target CAFs and gastric cancer cells while minimising damage to normal cells [19]. Oncolytic adenovirus‐based gene therapy (e.g., OBP‐702) and low‐immunogenic FAP‐CAR‐T cells effectively reduce CAF populations and tumour burden. Smart CAR‐T cells targeting both FAP+ CAFs and tumour‐associated antigens limit off‐target effects. Simultaneously, nanotechnology advancements have revolutionised drug delivery strategies targeting CAFs. Liposomes and nanoparticles (NPs) can precisely target CAFs, inhibiting their bioactivity. Gold nanoparticles (GNPs) enhance targeted drug release precision, promoting the transition of CAFs from the activated to the resting state. Building upon these approaches, innovative therapeutic strategies targeting CAFs hold great promise for improving gastric cancer treatment [20].

## 9 Conclusion

CAFs are a core component of TME in gastric cancer, influencing invasion, metastasis, and drug resistance. We systematically elucidated the key mechanisms of CAFs in gastric cancer progression from multiple perspectives, including metabolic reprogramming, extracellular matrix remodelling, epigenetic regulation, spatiotemporal dynamics, and pro‐cancer and drug‐resistant signalling pathways between CAFs, gastric cancer cells, tumour stem cells, and immune cells. The heterogeneity of CAFs and dynamic interactions with cancer cells pose challenges but also offer opportunities for new therapies. Advanced technologies like single‐cell sequencing and spatial transcriptomics have deepened the understanding of CAF markers and their dynamic changes, offering new possibilities for precise therapeutic targets. Additionally, elucidating the molecular mechanisms of CAF‐gastric cancer cell interactions, especially their role in therapeutic resistance, will be a key future research direction. Innovative strategies, including gene editing and nanotechnology, show promise in preclinical studies, though clinical translation remains challenging. Future research will focus on elucidating CAF‐cancer cell interactions and optimising therapeutic approaches.

## Author Contributions

Shiyang Deng, Yong zhen Chen, and Jiang Du designed and wrote the manuscript.

## Conflicts of Interest

The authors declare no conflicts of interest.

## Data Availability

The authors have nothing to report.
